# An upper arm ganglion cyst connected to the bicipital groove associated with glenohumeral osteoarthritis: a case report

**DOI:** 10.1016/j.xrrt.2024.01.009

**Published:** 2024-02-16

**Authors:** Ryosuke Tsujisaka, Noboru Matsumura, Yusaku Kamata, Hideo Morioka, Yasuhiro Kiyota, Taku Suzuki, Takuji Iwamoto

**Affiliations:** a Department of Orthopedic Surgery, Keio University School of Medicine, Tokyo, Japan; b Department of Orthopedic Surgery, National Hospital Organization Tokyo Medical Center, Tokyo, Japan

**Keywords:** Ganglion cyst, Upper arm, Bicipital groove, Glenohumeral arthritis, Long head of biceps brachii, Reverse shoulder arthroplasty

Ganglion cysts are articular or tendon-sheath lesions, caused by the herniation of the synovium or tendon fibrils. They commonly affect women aged 30-50 years. They most frequently develop in the wrist region and are occasionally associated with trauma.^5^ Although several studies have reported ganglion cysts around the shoulder in the spinoglenoid or scapular notch,^3^^,^^4^ ganglion cysts occurring around the long head tendon of the biceps brachii (LHB) are rare. This study reports a case of an upper arm ganglion cyst arising from the bicipital groove, associated with glenohumeral osteoarthritis, that was successfully treated with reverse shoulder arthroplasty (RSA).

The patient was informed that the data concerning the case would be submitted for publication, and she provided consent.

## Case report

An 84-year-old female had gradually worsening left shoulder pain for two years. One month prior, the patient suddenly experienced upper arm pain and noticed an enlarging mass on the upper arm. She was referred to our institution with a chief complaint of a painful mass on the left upper arm.

**Figure 1:**
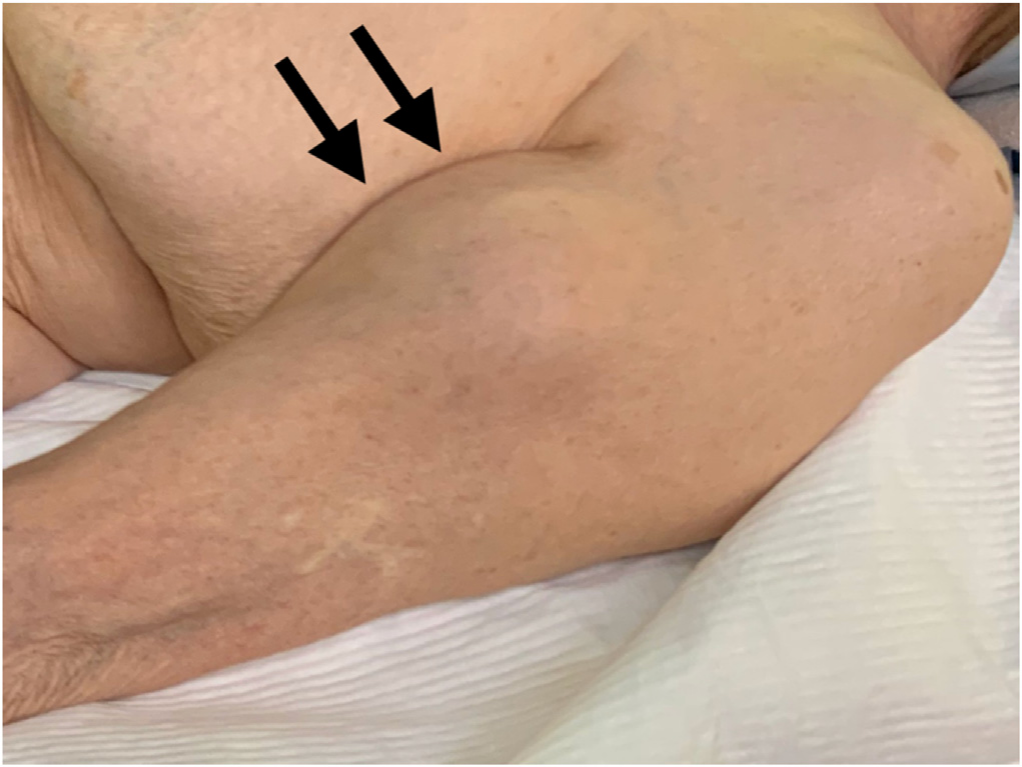
The appearance of the mass over the mid-anterior aspect of the upper left arm.

**Figure 2:**
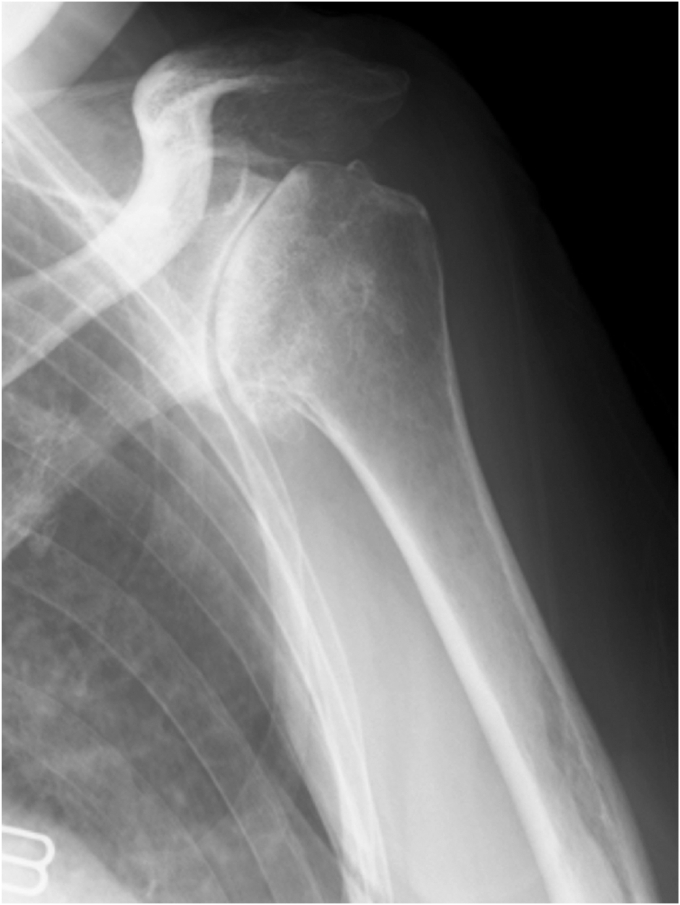
Preoperative radiographs of the left shoulder showed glenohumeral joint space narrowing, subchondral sclerosis, and osteophytes on the inferior humeral head.

**Figure 3:**
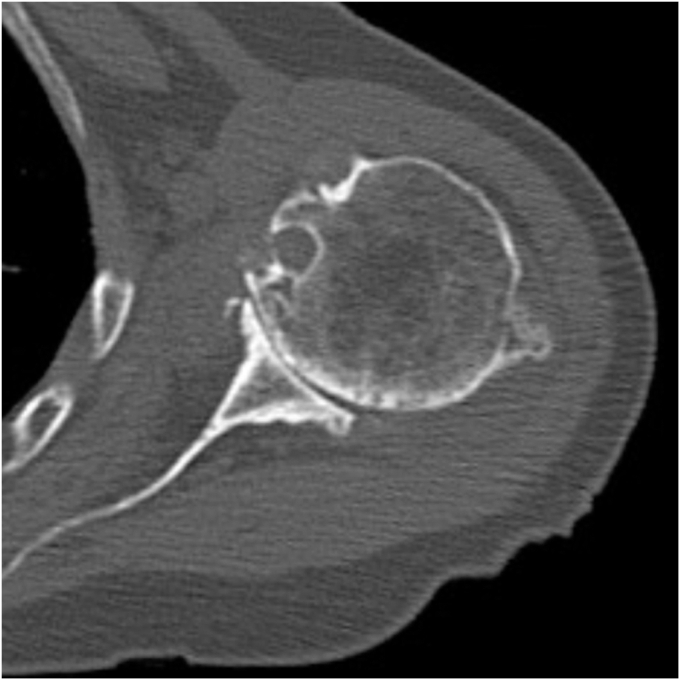
Preoperative computed tomography scans showed subchondral sclerosis of the glenohumeral joint and osteophytes around the bicipital groove.

**Figure 4:**
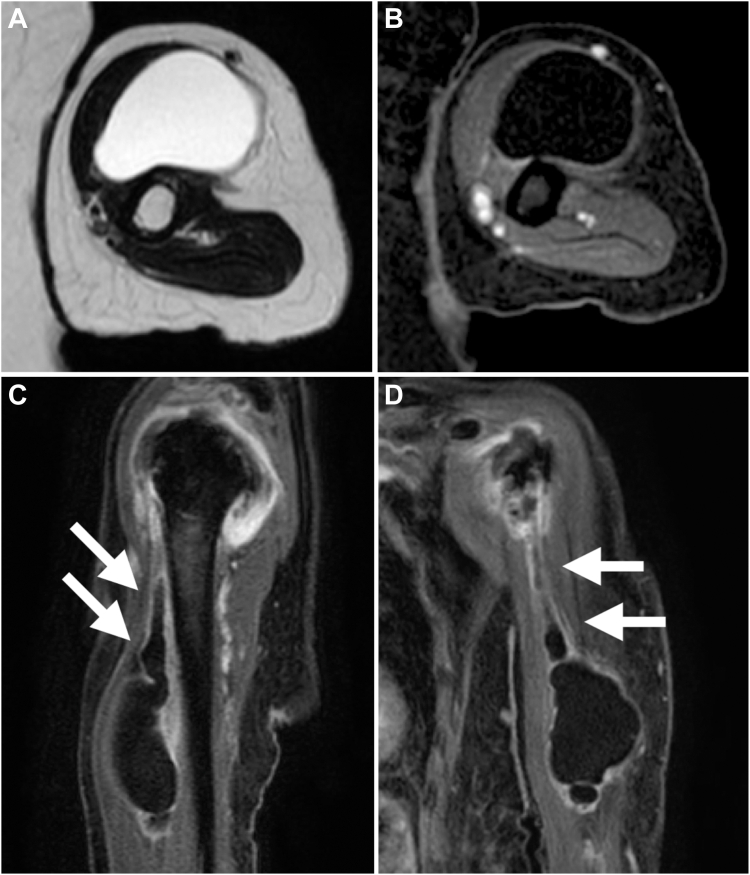
Preoperative magnetic resonance images of the left upper arm. (**A**) Axial images showed a homogenous left upper arm mass, measuring 6 × 4 cm, with a hyperintense signal on T2-weighted imaging. (**B**) The mass exhibited a hypointense signal on fat-suppressed T1-weighted imaging and was not enhanced. (**C**) Sagittal images showed a proximal extension of the mass. (**D**) Coronal images revealed that the proximal end of the mass was connected to the bicipital groove along with the long head tendon of the biceps brachii muscle.

Physical examination revealed a tender mass, measuring 6 × 4 cm, on the mid-anterior aspect of the left upper arm (Fig. 1). She experienced severe pain in the shoulders and upper arm at night and during arm movements. Active shoulder motion was restricted to 80° in anterior elevation, 45° in abduction, 10° in external rotation with the arm on the side, and up to the buttocks for internal rotation behind the back on the affected side. Anteroposterior radiography of the shoulder revealed narrowing of the joint space and osteophytes in the inferior humeral head (Fig. 2). Computed tomography revealed subchondral sclerosis of the glenohumeral joint and osteophytes around the bicipital groove (Fig. 3). Magnetic resonance imaging showed a homogenous mass, measuring 6 × 4 cm, extending from the bicipital groove. It exhibited a hyperintense signal on T2-weighted imaging (Fig. 4, *A*) and a hypointense signal on T1-weighted imaging (Fig. 4, *B*). The mass was not enhanced, and the proximal end of the mass was connected to the bicipital groove along the LHB (Fig. 4, *C* and *D*). There was no evidence of a rotator cuff tear. Ultrasound-guided aspiration of the mass revealed approximately 100 mL of pale-yellow viscous drainage, and cytology showed no malignant findings, consistent with a ganglion cyst. However, the mass recurred two weeks later. The patient was diagnosed with a recurrence of an upper arm ganglion cyst arising from the bicipital groove, associated with primary glenohumeral osteoarthritis. Since the patient had difficulty performing daily activities due to the painful upper arm mass and limited glenohumeral joint motion, she was scheduled to undergo an RSA.

**Figure 5:**
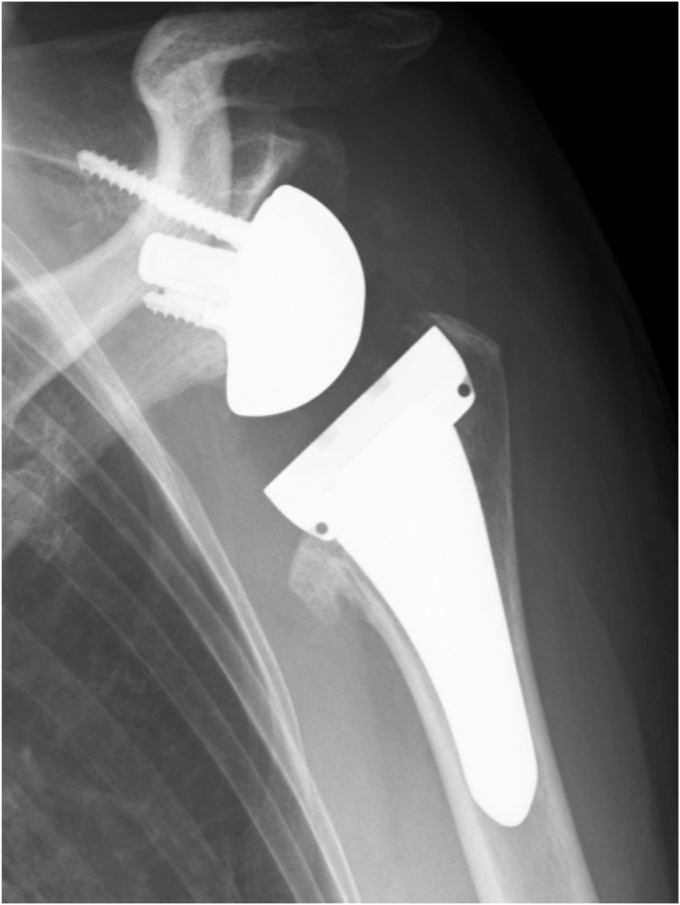
Postoperative radiograph two years after reverse shoulder arthroplasty.

Under general anesthesia, RSA (Medacta Shoulder System; Medacta International, Castel San Pietro, Switzerland) was performed via the deltopectoral approach in a beach chair position. When the bicipital groove was exposed and the transverse humeral ligament was sectioned, a flattened LHB with pale-yellow jelly drainage was observed. The LHB was cut at the level of the bicipital groove and tied to the pectoralis major tendon. No additional treatment was administered to the upper arm mass. The subscapularis tendon was peeled from the lesser tuberosity, and the humeral head was cut at the level of the anatomical neck. The 27-mm baseplate was fixed with four screws in the neutral version, and a 36-mm glenosphere was selected. The humeral short stem and metaphysis were inserted at 20° retroversion. After RSA reduction, the subscapularis tendon was repaired using three polyethylene sutures (#2 FiberWire; Arthrex, Inc, Naples, FL, USA) (Fig. 5).

**Figure 6:**
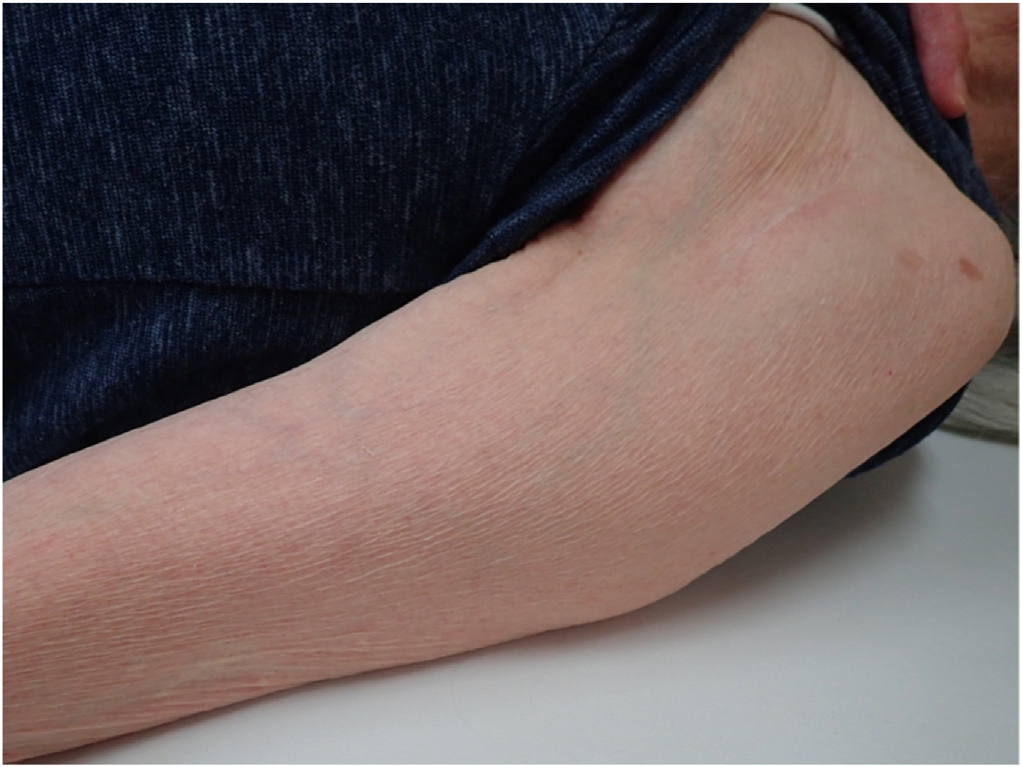
During the final follow-up two years postoperatively, no recurrence of the upper arm mass was detected.

The upper arm mass disappeared immediately after implantation. The shoulder was immobilized with a brace for four weeks. Stooping, pendulum, and passive shoulder range of motion exercises were started one week postoperatively. Active shoulder motion was allowed four weeks after surgery. During the final follow-up two years after surgery, no recurrence of the mass was observed (Fig. 6). The active range of motion of the left shoulder was 130° for anterior elevation, 50° for external rotation, and up to the 12th thoracic vertebral level for internal rotation behind the back. The American Shoulder and Elbow Surgeons shoulder score improved from 20 preoperatively to 67 at the final follow-up.

## Discussion

The present case involved a painful mass in the upper arm and glenohumeral osteoarthritis. The synovial fluid escaped from the glenohumeral joint through the bicipital groove and communicated with the upper arm mass. On magnetic resonance imaging, the proximal end of the mass was connected to the bicipital groove. Intraoperatively, there was a degenerated and flattened LHB. These findings indicated that the space in the bicipital groove was narrowed by the flattened LHB, and it acted as a one-way check valve for ganglion cysts. The hypertrophic portion of the LHB reportedly caused hourglass biceps, wherein the LHB is entrapped and unable to slide into the bicipital groove.^1^ In the present case, the pathology was caused by a mechanism similar to the hourglass biceps phenomenon. Two case reports of cystic lesions around the LHB have been published^2^^,^^6^. However, these cases involved the glenohumeral joint and were associated with rotator cuff tears resulting from exercise or trauma. The underlying mechanism of the development of a cyst in the present case differed from that of the previous cases.

Resection of the mass was a viable treatment option in this case. However, when cyst formation persists, the mass may recur. LHB tenotomy or tenodesis, which excludes the one-way check valve mechanism, was another option. However, the patient also experienced limited motion and pain in the shoulder joint due to glenohumeral arthritis. Thus, the glenohumeral joint was replaced in the present case. Following shoulder replacement, synovial fluid due to glenohumeral joint degeneration was not detected. Furthermore, the check valve mechanism was excluded by resecting the LHB in the bicipital groove. A recent study revealed that the clinical outcomes of patients who underwent RSA were compatible with those who underwent anatomical total shoulder arthroplasty, among elderly patients with glenohumeral osteoarthritis without rotator cuff tears.^7^ Since postoperative rotator cuff deficiency and degeneration were concerns in the present case, RSA was selected for the 84-year-old patient. Although the cyst was not removed, no recurrence of the mass was observed two years postoperatively. RSA successfully improved shoulder function and relieved the upper arm pain, which was related to the upper arm ganglion cyst.

This study reported a case of shoulder osteoarthritis with a ganglion cyst in the upper arm around the LHB. Ganglion cysts along the LHB are likely caused by shoulder osteoarthritis. RSA was a viable initial surgical option for treating primary shoulder arthritis in the elderly.

## Conclusion

An 84-year-old female with glenohumeral osteoarthritis presented with a large painful mass in the left upper arm, connected to the bicipital groove. RSA successfully alleviated the pain and limited motion without the recurrence of the mass. An upper arm ganglion cyst arose from the glenohumeral joint through the bicipital groove. Shoulder arthroplasty inhibited the production of synovial fluid in the glenohumeral joint, and the one-way check valve of the cyst at the bicipital groove was removed.

## Disclaimers:

Funding: No funding was disclosed by the authors.

Conflicts of interest: The authors, their immediate families, and any research foundation with which they are affiliated have not received any financial payments or other benefits from any commercial entity related to the subject of this article.

Patient consent: Obtained.
